# From ‘glass basins’ to ‘glass barriers’

**DOI:** 10.1093/nsr/nwae157

**Published:** 2024-04-30

**Authors:** Yuliang Jin

**Affiliations:** Institute of Theoretical Physics, Chinese Academy of Sciences, China; School of Physical Sciences, University of Chinese Academy of Sciences, China; Center for Theoretical Interdisciplinary Sciences, Wenzhou Institute, University of Chinese Academy of Sciences, China

**Figure 1.**
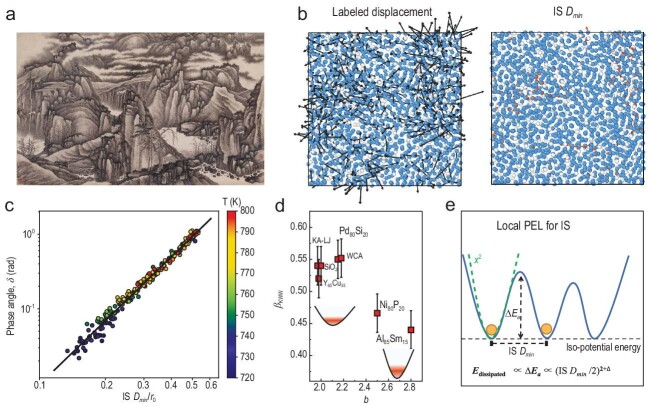
(a) Thousands of peaks and myriads of valleys, by Gong Xian (Qing Dynasty), in the collection of the Museum Rietberg (image source: Wikimedia Commons). (b) The left panel shows typical particle displacements (black arrows) in a glass-forming liquid and the right panel shows the corresponding IS *D*_min_ displacements (red arrows). (c) Scaling relation between δ and IS *D*_min_ in Al_85_Sm_15_ (see Equation (1)). (d) Correlation between β_KWW_ and *b*. (e) Schematic potential energy landscape (PEL) visualizing the relationship between the basin distance ${\rm IS} \, D_{\rm min}$ and the energy barrier Δ*E_a_*. Panels (b–e) are adapted from ref. [8].

If a supercooled liquid is cooled rapidly to avoid crystallization, a glass is formed. During this procedure, the viscosity can increase by more than 10 orders of magnitude over a small temperature range near the glass transition temperature *T*_g_, while the particle configuration remains disordered [1]. Understanding the nature of glasses is a key challenge in modern solid-state physics and statistical mechanics. A useful description of glasses is provided by the energy landscape, representing the dependence of the potential energy on particle coordinates [2]. Such a landscape generally consists of enormous metastable ‘glass basins’ corresponding to local energy minima (also called inherent structures) and ‘glass barriers’ separating basins—just as we often see in a traditional Chinese landscape painting (Fig. 1a). The hierarchical organization of glass basins was mathematically established by Giorgio Parisi using replica theory [3]: there are many basins in the energy landscape, and each basin splits into multiple sub-basins, which further split into smaller sub-sub-basins, etc. This peculiar behavior is termed *full-step replica symmetry breaking* in spin glasses [4] and a *Gardner transition* in structural glasses [5,6]. As a consequence of hierarchical basins, the inherent structures are embedded in an ultrametric space: the distance $\mathcal {D}$ between any three inherent structures *a, b* and *c* satisfies the inequality $\mathcal {D}(a,c) \le \rm {max}[\mathcal {D}(a,b), \mathcal {D}(b,c)]$, where the equality holds only if *a, b* and *c* are at the same level in the basin hierarchy. What remains to be systematically explored are statistics of glass barriers and properties of associated saddle points, which have attracted great interest in recent years [7].

In a recent study published in *National Science Review*, Yu *et al.* [8] proposed a new measure of the distance between inherent structures, and revealed its universal correlation to energy barriers. Conventionally, the distance between two configurations is measured by particle displacements with particle indexes fixed; see the left panel of Fig. 1b. A new distance parameter, called the *inherent structure minimal displacement* (${\rm IS} \, D_{\rm min}$), was introduced in [8] to quantify the minimal cooperative rearrangements between two inherent structures, based on a pattern-matching technique allowing particle reindexing; see the right panel of Fig. 1b. A power-law scaling is established between ${\rm IS} \, D_{\rm min}$ and the loss angle δ quantifying the energy dissipation during cyclic shear perturbations:


(1) \begin{eqnarray*}
\delta \sim ({\rm IS} \, D_{\rm min})^b.
\end{eqnarray*}

Scaling Equation (1) is observed in seven simulated model glass formers, including four metallic glass models (Al_85_Sm_15_, Ni_80_P_20_, Pd_80_Si_20_ and Y_65_Cu_35_), the Kob-Anderson model based on the Lennard-Jones force field (KA-LJ), the Weeks-Chandler-Anderson (WCA) model and a silica (SiO_2_) model, independent of the temperature and pressure (Fig. 1c). The exponent *b* ∼ 2 depends modestly on the model, and is correlated to the stretching exponent β_KWW_ in the Kohlrausch-Williams-Watts (KWW) decay $f(t) \sim \exp [(-t/\tau )^{\beta _{\rm KWW}}]$ of the loss shear modulus *G*″(*t*) (Fig. 1d).

The loss angle δ can be interpreted as a parameter of the energy damping due to activated processes over a typical energy barrier Δ*E_a_*. Equation (1) then provides a relationship between the barrier height Δ*E_a_* and the basin distance ${\rm IS} \, D_{\rm min}$, $\delta \sim \Delta E_a \sim ({\rm IS} \, D_{\rm min})^b$. An exponent *b* = 2 is expected for harmonic basins, and the deviation Δ = *b* − 2 describes the anharmonic effects (Fig. 1e).

In summary, this study provides new insight into the complex structure of the energy landscape in glass materials. The universal connection between glass basins and glass barriers has the potential to deepen our understanding of activated dynamics in disordered systems [9], and the statistics of plastic events in amorphous solids [10].
